# Supplementary material for: Acceptance and compliance with micronutrient powder (MNP) among children aged 6–23 months in northern Nigeria
Source: PLOS Glob Public Health. 2022 Oct 17;2(10):e0000961. doi: 10.1371/journal.pgph.0000961 (PMC10022258; doi:10.1371/journal.pgph.0000961)
Supplement: S1 Dataset — (ZIP) [file pgph.0000961.s008.zip › S8_Dataset/Direct Observations.zip!/Direct Observations/190605_A_SO_AD_1_DO.pdf]

1.1.1 Instrument F –Direct Observation Form

<<Instrument F Direct Observation Form>>

PI Name: PragyaMathema,Stephen Kodish
Study Title: Formative research to develop an infant and young child feeding (IYCF) intervention with micronutrient powder (MNP) for children aged 6 – 23 months
PI Version/Date: Version1.0/January 24, 2015

Date: _June 19, 2015____________Location: __Bolki community, Song LGA_________

Data Collector: Aisha Dahiru______________Participant ID: __Mud House with zinc Door_______

Main goals of this direct observation are to answer the following questions:

1. In this household, what would be the best way(s) to give nutritional supplements to young children? Why?

2. In this household, describe the food sharing that you observe throughout the meal. Are there any predictable patterns?

3. In this household, who is favored and how is this favoritism expressed during meal preparation and eating?

(TEAR OFF AT PERFORATED DOTTED LINE BELOW)

PARTICIPANT ID: _________

Household Composition Table (Fill out as you do data collection)

INITIALS	SEX (M/F)	AGE (estimated)	Relationship to household	Description
M	F	27YRS	Mother	Wearing a brown shirt and has facial mark.
G	F	17 MONTHS	BABY GIRL	Wearing green dress
B	M	6 YEARS	BOY	Wearing yellow checked shirt.
OG	F	4 YEARS	OLDER GIRL	Wearing yellow checked shirt with green skirt
GF	F	5 YEARS	OLDER GIRL FRIEND	Wearing purple dress
MF	F	29 YEARS	MOTHER’S FRIEND	Wearing brown wrapper with green shirt and head tie

PARTICIPANT ID: __________

TIME	ACTOR INITIALS	RECIPIENT INITIALS	WHAT HAPPENED	FOOD	Reactivity
8.00	M		Sweeping		
8:05	M		Sweeping		
8:10	M		Sweeping		
8:15	M		Washing utensils		
8:20	M		Washing utensils		
8:25	M		Washing utensils		
8:30	M		Washing utensils		
8:35	M	G	Bathing the baby girl		
8:40	M	G	Bathing the baby girl		
8:45	M	G	Dressing the baby girl		

TIME	ACTOR INITIALS	RECIPIENT INITIALS	WHAT HAPPENED	FOOD	Reactivity
8:50	M		Serving kunu from the flask into a cup	Kunu gyada (ground nut gruel)	
8:55	M		Mixing MNP into a small portion of the kunu	Kunu gyada (ground nut gruel)	
8:56	M	G	Mother is feeding he baby with the portion of kunu mixed with mnp	Kunu gyada (ground nut gruel)	
8:57	M	G	Feeding the baby		
8:58	M	B	Feeding the baby		
8:59	M	B	Feeding the baby		
9:00	M	B	Feeding the baby		
9:01	W	B	Cleaning the baby mouth		
9:02	M	B	Picks hoes and a container		
9:03	M	B	Carrying the baby on her back		
9:04	M		Going to the farm with the baby on her back		
9:05	M	B	Place the baby on the ground		
9:10	M	B	Farming		
9:15	B		Playing on the farm		
9:20	B		Playing		
9:30	M	B	Breastfeeding the baby		

TIME	ACTOR INITIALS	RECIPIENT INITIALS	WHAT HAPPENED	FOOD	Reactivity
9:35	M	B	Breastfeeding the baby		
9:40	M	B	Breastfeeding the baby		
9:45		B	Sleeping		
9:50	M	B	Lying baby on a wrapper in the farm		
9:55	M		Farming		
10:00	M	G	Carrying baby on the back and carrying container on head		
10:05	M		Walking home		
10:10	M	G	Holding the baby		
10:15	B		Sleeping in mother's arms		
10:20	M	G	Lying baby on the mat		
10:25	M	G	Loosening the baby's hair		
10:30	M	G	Loosening the baby's hair		
10:35	M	G	Rubbing oil in baby's hair		
10:40	B		Boy sitting beside mother with his book		
10:45	M		Plaiting the baby's hair		
10:50	M		Plaiting the baby's hair		
10:55	M		Plaiting the baby's hair		
11:00	M		Plaiting the baby's hair		
11:05	G		Sleeping		
11:10	G		Baby Sleeping		
11:15	M	G	Plaiting the baby's hair		

TIME	ACTOR INITIALS	RECIPIENT INITIALS	WHAT HAPPENED	FOOD	Reactivity
11:20	B		Boy goes out chatting with his friends		
11:25	G		Baby girl wakes up		
11:30	G		Baby girl lying on mothers lap		
11:31	M	B	Mother breastfeeding baby girl		
11:32	M	G	Mother breastfeeding baby girl		
11:33	M	G	Mother breastfeeding baby girl		
11:34	M	G	Mother breastfeeding baby girl		
11:35	M	G	Mother breastfeeding baby girl		
11:40	M	G	Mother breastfeeding baby girl		
11:45	M	G	Mother breastfeeding baby girl		
11:50	G		Baby girl is playing		
11:55	M		Takes a bag to the kitchen		
12:00	M		Plucking leaves from a tree inside the house		
12:00	G		Baby girl is playing with the leaves her mother was plucking		
12:00	M		Mother feeds the sheep with the leaves plucked		
12:05	M		Mother looks on as the sheep eats the leaves		
12:05	G		Baby girl playing around the sheep shade		
12:10	G		Baby girl playing around the sheep shade		
12:10	M		Cleaning the sheep shape		
12:15	M		Cleaning the sheep shape		

TIME	ACTOR INITIALS	RECIPIENT INITIALS	WHAT HAPPENED	FOOD	Reactivity
12:15	G		Baby girl squatting to urinate near the sheep shade		
12:20	M	G	Washing the baby hands and foot		
12:25	M	G	Mother helps the baby wear her panties		
12:30	G		Baby girl playing around the house	R	
12:50	M		Baby girl playing around the house		
12:50	G		Mother brings out vegetable	Sesame leaves	
12:55	G		mOther cutting the Sesame leaves into a large bowl	Sesame leaves	
01:00	M		mother cutting the Sesame leaves into a large bowl		
01:05	G		Baby sits beside the mother		
01:10	M		baby playing with the sesame leaves	Sesame leaves	
01:15	B		Mother cutting the sesame leaves in a large bowl	Sesame leaves	
01:20	B		Playing with the sesame leaves		
01:25	M	G	Playing with the sesame leaves		
01:30	M	G	Mother Breastfeeding baby while cutting the leaves		
01:35	M	G	Mother Breastfeeding baby		
01:40	M		Mother goes into the kitchen to pick drying mat		
01:45	G		Baby follow the mother around the house		
01:50	M		Mother spreading the sesames leaves on the mat		
01:55	B		Baby playing with the leaves on the mat		
02:00	OG		Older girl comes back from school		
02:05	OG		Older girl changes uniform and and		

TIME	ACTOR INITIALS	RECIPIENT INITIALS	WHAT HAPPENED	FOOD	Reactivity
			wear dress		
02:10	M		Mother lying down under the shade with baby girl		
02:15	B		Boy joins mother and baby girl under the shade		
02:20	OG		Older girl washing her hands		
02:25	OG		Older girl bring out food from the kitchen to shade	Jollof rice	
02:30	M		Mother serves kunu gyyada Into a large bowl	Kunu gyada	
02:30	M		Mother adding sugar to kunu	Kunu gyada	
02:30	M		Serving food in two separate bowls	Jollof rice	
02:31	M		Gives a bowl to older girl and boy		
02:32	OG		Eating together with boy	Jollof rice	
02:33	B		Eating together with older girl	Jollof rice	
02:34	G		Eating with her hand from separate bowl	Jollof rice	
02:35	G		Baby girl eating by herself from a separate bowl	Jollof rice	
02:36	G		Baby girl eating by herself from a separate bowl	Jollof rice	
02:37	M	G	Baby girl protesting when the mother tries to feed her		
02:38	OG	G	Baby girl protesting when the older girl tries to feed her		
02:39	B	G	Baby girl protesting as the boy tries to feed her		
02:40	G		Baby girl eating by herself from a separate bowl		
02:41	G		Baby girl eating by herself from a separate bowl		
02:42	G		Baby girl eating by herself from a separate bowl		
02:43	G		Baby girl eating by herself from a		

TIME	ACTOR INITIALS	RECIPIENT INITIALS	WHAT HAPPENED	FOOD	Reactivity
			separate bowl		
02:44	G		Baby girl eating by herself from a separate bowl		
02:45	M		Mother serving kunu into separate cups		
02:46	B		Drinking kunu from separate cup	Fish	
02:47	OG		Drinking kunu from separate cup		
02:48	G		baby girl drinking kunu from separate cup by herself		
02:49	G		Mother serving kunu to older girl friend		
02:50	G		Crying over older girl friend cup of kunu		
02:50	M		Mother handing baby girl empty cup		
02:51	G		Drinking kunu herself from separate cup		
02:52	G		Older girl washing baby girl hands and mouth		
02:53	B		Washing his hands		
02:54	B		Washing his hands		
02:55	OG		Washing the dishes they just used	Water	
02:55	B		Washing dishes		
02:56	G	B	Playing with older boy and girl friend		
02:57	G	B	Walks out of the house		
03:02	G		Baby girl playing outside the house with other children		
03:07	G		Baby girl playing outside the house with other children		
03:12	G		Baby girl playing outside the house with other children		
03:17	G		Baby girl playing outside the house with other children		
03:22	G		Baby girl playing outside the house with other children		
03:32	G		Baby girl playing outside the house with other children		

TIME	ACTOR INITIALS	RECIPIENT INITIALS	WHAT HAPPENED	FOOD	Reactivity
03:37	G		Going back to the house		
03:42	G	M	Baby girl playing with mother		
03:47	M	G	Mother giving baby water		
03:57	B	M	Older brother playing with baby girl		
04:02	M	G	Breastfeeding baby girl		
04:07	G	M	Mother breastfeeding the baby girl		
04:12	M	G	Boy lying down beside down beside mother under shade		
04:17	M	G	Walking into the house		
04:27	G		Mother chatting with her friend		
04:27	G	B	Walk into the room to pick MNP sachet		
04:27	B		Mother hands the MNP packet to her friend		
04:27	G	B	Mother chatting with her friend about MNP		
04:32	B		Baby girl Playing with older boy		
04:37	M		Mother chatting with her friend		
04:42	M		Mother breast feed the girl while chatting with her friend		
04:47	G		Baby girl lying down on mother		
04:47	B		Older brother lying beside mother		
04:52	M	MF	Mother chatting with her friend		
04:57	M	MF	Mother chatting with her friend		
05:02	MF	M	Mother's friend bids her goodbye		
05:07	M	G	Mother bathing baby girl		
05:12	M	G	Mother bathing baby girl		
05:17	M	G	Mother bathing baby girl		
05:22	M	G	Mother rubbing baby girl oil		
05:27	M	G	Mother dressing baby girl		
05:32	M	G	Mother carrying her on her back		
05:37	M	G	Mother carrying her on her back		
05:42	M	G	Mother lying her down on the shade		
05:47	M		Mother sweeping the compound		
05:52	M		Mother sweeping the compound		R
05:57	M	G	Mother checking on the baby		
06:02	M		Mother lying down beside the baby		

TIME	ACTOR INITIALS	RECIPIENT INITIALS	WHAT HAPPENED	FOOD	Reactivity

TIME	ACTOR INITIALS	RECIPIENT INITIALS	WHAT HAPPENED	FOOD	Reactivity

PARTICIPANT ID: __Mud house with zinc door_______

Primary Questions (Complete <u>after</u> you complete data collection):

From what you have seen observing THIS HOUSEHOLD, please answer the following questions in detail:

Question	Write your <u>detailed</u> response here
1. In this household, what would be the best way(s) to give nutritional supplements such as an MNP to young children to ensure compliance?<br><br>Why?	The best way to ensure compliance is to educate the mother on MNP because she is the one taking care of the baby and feed the baby in a separate bowl
2. In this household, who is favored during meal preparation or feeding?	The older children Were favored during feeding .<br>The baby ate in a separate bowl

How is this favoritism expressed?	The food was in solid state.
3. How could we promote the MNP supplement for this family that would attract their attention?<br><br>Who would they listen to?	MNP can be promoted to this family through the mother .<br>The father ca also serve as an influence because he might be the one responsible for all decisions in the household .<br>The religious leaders in the community can also have influence because the mother was discussing how their religious leader influenced acceptance of other products that were previously introduced into the community
4. In this household, what would be the best ways to promote the MNP but limit how much it would be shared?<br><br>Why?	The best way to limit the sharing of MNP is to ensure the mother understands that MNP is meant for children under 2 years and should be given to the child separately.

5. What other issues and concerns did you see with this family that are important for us to consider with regard to introduction of an MNP?<br><br>Why?	The young child was eating by herself. The food given to the young child was solid.
6. Based on your experience observing this child today, do you believe reactivity negatively impacted your ability to see the food-related behaviors of the child?<br><br>Please explain your answer in detail.	Reactivity did not impact their food related behavior .
